# Utilization and Cost of Inpatient Dermatologic Procedures: A Cross-sectional Analysis

**DOI:** 10.7759/cureus.4586

**Published:** 2019-05-02

**Authors:** Kory P Schrom, Raghav Tripathi, Harib H Ezaldein, Jeffrey F Scott

**Affiliations:** 1 Dermatology, University Hospitals Cleveland Medical Center, Cleveland, USA; 2 Dermatology, Case Western Reserve University, Cleveland, USA

**Keywords:** inpatients, outpatients, cost, dermatologic procedures, admissions, payers

## Abstract

Knowledge surrounding inpatient dermatologic procedure costs is limited; therefore to learn more, we performed a cross-sectional analysis of dermatologic procedures contained in a publicly available Washington State Comprehensive Hospital Abstract Reporting System database from 2014. Dermatologic procedure utilization and cost were evaluated based on several parameters including demographics, length of hospital stay, payments, and payers. SAS 9.4 was used for the analysis. A total of 14,768 patients underwent dermatologic procedures in 2014 and 81.0% were white. The average age was 53 years (SD = 0.17), and the average payment for all patients who underwent dermatologic procedures was $85,059.48 (SD = $1,284.34). The average hospital length of stay was 8.91 days (SD = 0.07). The most common admission type was elective (66.2%), the most common admit source was a non-healthcare facility point of origin (78.2%), the most common primary payer was Medicare (36.2%), and the most common procedure was incision and drainage of skin and subcutaneous tissue (26.5%), followed by closure of skin and subcutaneous tissue of other sites (20%). This analysis demonstrated that inpatient dermatologic procedures are a significant driver of inpatient health care costs, and it is critical to determine factors that increase inpatient costs related to dermatologic procedures in order to develop strategies for reducing healthcare costs.

## Introduction

Health care spending accounted for 17.9% of the nation’s gross domestic product (GDP) in 2017, or $3.5 trillion [1]. The contribution of inpatient dermatologic procedures to this total is poorly understood. For example, the cost of dermatologic procedures among Medicare beneficiaries was $2.21 billion in 2017, with a portion arising from inpatient care [2]. We examined discharge records from community hospital visits to better understand the utilization and cost of non-disease-specific inpatient dermatologic procedures.

## Materials and methods

We performed a cross-sectional analysis of the Washington State Comprehensive Hospital Abstract Reporting System (CHARS) database to analyze the utilization and cost of inpatient dermatologic procedures performed in 2014 [3]. This database contains record-level data pertaining to inpatient and observation community hospital visits and abstracted information on discharges for civilian hospitals in the state. CHARS database collection methods have been described elsewhere [4]. This study was IRB exempt due to the de-identified and publicly available data.

All patients were adults (>18 years of age) hospitalized in Washington in 2014 who underwent a variety of dermatologic procedure. Descriptive analyses were performed on patient demographics, length of hospital stay, admission type, payment amount, and primary payer. All statistical analyses were performed using SAS 9.4 (Cary, NC).

## Results

A total of 14,768 patients underwent dermatologic procedures in 2014. Descriptive characteristics of the sample are provided in Table 1. The majority of admissions were elective (66.2%). The most common primary payer was Medicare (36.2%) followed by Medicaid (28.5%). The mean total hospitalization payment for patients undergoing dermatologic procedures was $85,059.48, and the average length of hospital stay was 8.9 days. The most common procedure was incision and drainage of skin and subcutaneous (SC) tissue (26.5%).

**Table 1:** Characteristics of patients undergoing an inpatient dermatologic procedure N, number; HMO, health maintenance organization; USD, United States dollar; SC, subcutaneous

Sample Size, N	14,768
Age (years), mean (SD)	52.6 (0.17)
Payment (USD), mean (SD)	$85,059.48 ($1,284.34)
Length of stay (days), mean (SD)	8.9 (0.07)
Sex, N (%)	
Female	6,560 (44.4)
Male	8,201 (55.6)
Race, N (%)	
Hispanic	812 (5.5)
White	11,965 (81.0)
Black	718 (4.9)
Other	1,273 (8.6)
Admit type, N (%)	
Elective	9,781 (66.2)
Emergency	2,071 (14.0)
Urgent	2,290 (15.5)
Other	626 (4.2)
Admit source, N (%)	
Non-healthcare facility point of origin	11,547 (78.2)
Clinic	1,346 (9.1)
Transfer from a hospital (different facility)	1,468 (9.9)
Other	407 (2.8)
Primary payer, N (%)	
Medicare	5,349 (36.2)
Medicaid	4,205 (28.5)
Health maintenance organization (HMO)	704 (4.8)
Commercial Insurance	2,575 (17.4)
Health Service Contractors	819 (5.6)
Other	1,116 (7.6)
Procedure, N (%)	
Skin & SC tissue aspiration	327 (2.2)
Injection or tattooing of skin lesion or defect	16 (0.1)
Incision & drainage of skin & SC tissue	3,891 (26.5)
Incision & removal of foreign body from skin and SC tissue	957 (6.5)
Incision of skin & SC tissue	199 (1.4)
Skin and SC tissue closed biopsy	539 (3.7)
Skin & SC tissue diagnostic procedures	2 (0.01)
Excisional debridement	1,918 (13.7)
Removal of nail anatomy	112 (0.8)
Skin chemosurgery	7 (0.1)
Dermal appendage ligation	35 (0.2)
Nail anatomy debridement	212 (1.4)
Non-excisional debridement	2,265 (15.4)
Skin & SC tissue closure	2,934 (20)

Characteristics of patients undergoing each of the identified procedures are provided in Table 2. Medicaid was the most common payer when incision and drainage of skin and SC tissue was performed. Skin chemosurgery had the highest average admission cost ($548,535.70) and excisional debridement had the highest aggregate cost ($5,333,677.80).

**Table 2:** Characteristics of dermatologic procedures USD, United States Dollar; SC, subcutaneous

	Skin & SC tissue aspiration	Injection or tattooing of skin lesion or defect	Incision & drainage of skin & SC tissue, other	Incision & removal of foreign body from skin and SC tissue	Incision of skin & SC tissue, other	Skin and SC tissue closed biopsy	Skin & SC tissue diagnostic procedures, other	Excisional debridement	Removal of nail anatomy	Skin chemosurgery	Dermal appendage ligation	Nail anatomy debridement	Non-excisional debridement	Skin & SC tissue closure, other sites
Total number of patients undergoing procedure	327	16	3,891	957	199	539	2	1,918	112	7	35	212	2265	2934
Age														
Mean	52.6	30.1	46.8	51.8	51.5	53.9	54	56.4	57.5	6.9	0.9	67.1	54.8	54.8
Std error	0.16	0.14	0.16	0.16	0.18	0.17	0.07	0.14	0.15	0.09	0.05	0.12	0.16	0.20
Length Stayed (Days)														
Mean	8.9	6.8	5.7	11.8	10.1	14.	5	10.8	9.9	47.4	5.7	16.9	11.5	7.2
Std error	0.10	0.10	0.07	0.21	0.12	0.18	0.01	0.12	0.09	0.49	0.12	0.15	0.15	0.09
Payment (USD)														
Mean	73,634.1	42,483.6	49,238.9	122,616.768	118,240.1	147,213.4	68,133.7	93,204	75,586.9	548,535.7	49,128.7	82,065.4	101,226.7	82,687.4
Min	3455	10855.9	1838	1699	7942.4	4176.3	68130.7	4248.3	7502.6	18255.9	1807.6	8343.3	2738.7	1,826.6
Max	1,158,397	172,812.3	2,248,144.1	4,586,665.2	982,935.6	362,0874.3	68,136.7	5,333,677.8	459,992.4	2,069,643.2	821,520.6	1,263,769.1	2,173,662.7	1,601,295.5
Std Err	50.1	106.5	12.4	69.6	97.3	112.1	0	35.4	71.2	2227.5	236.9	63.6	24.8	17.1
Sex														
Female	159	15	1718	548	85	258	0	819	41	3	15	73	926	1110
Male	168	1	2173	409	114	281	2	1099	71	4	20	139	1339	1824
Race														
Hispanic	13	2	199	50	10	36	0	6	0	5	10	108	175	8123
White	270	11	3177	743	151	411	1	1589	83	4	10	182	1866	2374
Black	20	1	202	57	11	21	0	74	14	0	8	11	126	126
Other	18	3	245	70	19	53	1	122	7	2	4	4	115	188
Admit Type														
Elective	237	8	3,100	522	108	314	2	1,200	80	5	0	101	1,423	2,116
Emergency	62	6	412	174	30	146	0	349	18	0	0	28	201	201
Urgent	24	1	361	251	59	7	0	348	13	1	1	83	150	150
Other	4	1	18	10	2	72	0	21	1	1	34	0	491	467
Primary Payer														
Medicare	123	1	1,036	394	68	202	0	839	51	0	0	132	872	1,114
Medicaid	90	9	1,701	185	61	130	1	482	30	5	19	32	561	648
Health Maintenance Organization (HMO)	22	0	175	45	11	18	0	91	5	0	1	22	119	114
Commercial Insurance	46	5	494	224	35	120	1	263	10	1	10	11	418	621
Health Service Contractors	19	0	185	69	11	45	0	114	8	0	2	10	115	105
Other	27	1	300	40	13	24	0	129	8	1	3	5	180	332

## Discussion

The majority (55.2%) of financial costs related to dermatologic care, including procedural costs, are experienced in the outpatient setting [5]. While inpatient costs contribute significantly to overall healthcare expenditures (20.1%), understanding of these costs is limited [1]. Moreover, data related to dermatologic costs in the inpatient setting primarily focus on disease-specific aggregate costs. For example, inpatient costs for care for psoriasis have been shown to be cost-prohibitive when compared to outpatient treatment (13,042 € versus 2,984 €), especially when accounting for biologic therapy use [6].

This current analysis is unique in that it identifies dermatologic procedures performed during an inpatient admission independent of dermatologic diagnosis and reveals potential factors that may be used to stratify patients undergoing a dermatologic procedure and suggest opportunities for cost reduction. This study is limited by its regional geographic encashment. Co-morbid diagnoses and direct cost of dermatologic procedures could not be analyzed, and aggregate cost reporting may have overestimated the relative cost contribution. Finally, provider specialty and level of training were not available.

Most patients received a dermatologic procedure during an elective admission and the primary payer was either Medicare or Medicaid. Additionally, our findings support dermatologic consultation when dermatologic procedures are considered. Diagnostic procedures recommended by an appropriately consulted dermatologist yield a definitive diagnosis in up to 80% of cases. Moreover, 45% to 80% of diagnoses will change after dermatology consultation preventing unnecessary therapy and costly hospital stays [7]. Finally, dermatology consultation may also facilitate outpatient follow-up for a procedure rather than performing it during admission.

## Conclusions

Inpatient dermatologic procedures are a significant driver of inpatient health care costs. It is critical to determine factors that increase inpatient costs related to dermatologic procedures to develop strategies for reducing healthcare costs. A good initial cohort for future study would be patients receiving a dermatologic procedure during an elective admission who have Medicare or Medicaid.
